# Chromosomal aberrations after induced pluripotent stem cells reprogramming

**DOI:** 10.1590/1678-4685-GMB-2020-0147

**Published:** 2021-09-03

**Authors:** Isadora May Vaz, Tamara Borgonovo, Tais Hanae Kasai-Brunswick, Danúbia Silva dos Santos, Fernanda Cristina Paccola Mesquita, Juliana Ferreira Vasques, Fernanda Gubert, Carmen Lúcia Kuniyoshi Rebelatto, Alexandra Cristina Senegaglia, Paulo Roberto Slud Brofman

**Affiliations:** 1 Pontifícia Universidade Católica do Paraná, Escola de Medicina, Núcleo de Tecnologia Celular, Curitiba, PR, Brazil.; 2 Universidade Federal do Rio de Janeiro, Instituto de Biofísica Carlos Chagas Filho, Rio de Janeiro, RJ, Brazil.; 3 Universidade Federal do Rio de Janeiro, Centro Nacional de Biologia Estrutural e Bioimagem, Rio de Janeiro, RJ, Brazil.; 4 Instituto Nacional de Ciência e Tecnologia em Medicina Regenerativa, Rio de Janeiro, RJ, Brazil.; 5 Instituto de Ciências Biomédicas, Rio de Janeiro, RJ, Brazil.

**Keywords:** Genetic instability, cytogenetics, iPSC, chromosomal aberration

## Abstract

Induced pluripotent stem cells (iPSCs) are generated from adult cells that have been reprogrammed to pluripotency. However, *in vitro* cultivation and genetic reprogramming increase genetic instability, which could result in chromosomal abnormalities. Maintenance of genetic stability after reprogramming is required for possible experimental and clinical applications. The aim of this study was to analyze chromosomal alterations by using the G-banding karyotyping method applied to 97 samples from 38 iPSC cell lines generated from peripheral blood or Wharton’s jelly. Samples from patients with long QT syndrome, Jervell and Lange-Nielsen syndrome and amyotrophic lateral sclerosis and from normal individuals revealed the following chromosomal alterations: acentric fragments, chromosomal fusions, premature centromere divisions, double minutes, radial figures, ring chromosomes, polyploidies, inversions and trisomies. An analysis of two samples generated from Wharton’s jelly before and after reprogramming showed that abnormal clones can emerge or be selected and generate an altered lineage. IPSC lines may show clonal and nonclonal chromosomal aberrations in several passages (from P6 to P34), but these aberrations are more common in later passages. Many important chromosomal aberrations were detected, showing that G-banding is very useful for evaluating genetic instability with important repercussions for the application of iPSC lines.

## Introduction

Induced pluripotent stem cells (iPSCs) have attracted attention because of their great potential for application in several areas of cell therapy. These cells can differentiate into any cell in the germ layers (endoderm, mesoderm or ectoderm). Due to the widespread use of iPSCs as a model to study diseases, mainly to understand their mechanisms, and their possible uses in regenerative medicine, iPSCs are believed to have a promising future (Ambrosio et al., 2015). iPSCs are currently being used for the study of many congenital syndromes, such as long QT syndrome (LQTS), which was the first heart disease model established using this cell type (Moretti et al., 2010).

Despite advances in stem cell culture conditions, an important issue that needs to be considered is the possibility of an increase in genomic instability. This could explain, at least in part, the potential of these cells to become tumorigenic (Catalina et al., 2007). In addition, altered karyotypes interfere not only with clinical applications but also with experimental results, such as in drug tests, because their sensitivity to drugs may be affected by aberrant expression patterns of the cells (Ben-David et al., 2014). The study of aneuploidies (numerical chromosomal alterations) is an important aim for guaranteeing the quality of these cells, especially when cells derived from somatic cells from patients have aged (Garcia-Martinez et al., 2016). In disease models, altered cells do not always reproduce the same characteristics as the normal cells that give rise to them (Ben-David *et al*., 2014), therefore, they cannot be used as a model. Thus, it is essential to guarantee the quality, safety and traceability of the cells.

The genomic integrity of the cells can be evaluated by a variety of techniques, including karyotype analysis (Mayshar et al., 2010; Ben-David et al., 2014). Many karyotypic abnormalities have been repeatedly reported in human iPSCs, such as trisomy 12 (Taapken et al., 2011; Ben-David *et al*., 2014; Lamm et al., 2016) and trisomy 17 (Ben-David and Benvenisty, 2011; Taapken *et al*., 2011; Dewhurst et al., 2014).

Through the G-banded technique, it is possible to detect numerical and structural anomalies, such as translocations and inversions. In addition to these advantages, this technique has a low cost and can detect low-level mosaicism (usually 20 metaphases are observed, which is likely to detect a result when more than 5%, 1 in 20, of cells present a chromosomal aberration) (Ben-David and Benvenisty, 2012). However, one of the limitations of the G-banding method is the impossibility of detecting some small alterations, thus complementary methods are sometimes required. The aim of this study was to analyze the genomic integrity of iPSCs using G-banded chromosomal karyotype analysis before and after genetic reprogramming and long-term cultivation to describe chromosomal instability in iPSCs from individuals with diseases associated with genetic mutations and in iPSCs of healthy individuals.

## Materials and Methods

### Patients and samples

The samples were separated into two groups: Group 1 was composed of samples from 21 patients with diseases associated with genetic mutations. These patients were diagnosed with long QT syndrome (LQTS) type 1 (mean age 22.2 years), LQTS type 2 (mean age 34.4 years), Jervell and Lange-Nielsen syndrome (JLNS) (mean age 3.5 years) or amyotrophic lateral sclerosis (ALS) (mean age 53.6 years), in which the source of the cells was peripheral blood. Group 2 was composed of samples from 17 healthy individuals, and the cells were obtained from peripheral blood (mean age 29.1 years) or Wharton’s jelly (a tissue present around the umbilical blood vessels).

### Cell culture and G-band karyotyping protocol

The human iPSC cell lines were generated from peripheral blood or from Wharton’s jelly at Universidade Federal do Rio de Janeiro. To generate the cell lines, a CytoTune ^TM^ 2.0- Sendai Reprogramming Kit containing OCT-3/4, Klf4, Sox2 and cMyc as reprogramming factors was used. Expression of pluripotency markers was determined by RT-PCR and flow cytometry (BD Accuri C6, BD FACSAria II and FlowJo software version 1). The reprogramming method used has already been validated (Kasai-Brunswick et al., 2018; Mesquita et al., 2019; Gubert et al., 2019a; Gubert *et al*., 2019b).

Confluence was evaluated before chromosomes were harvested. The ideal cytogenetic harvest condition was when the culture reached 60% to 80% confluence. Then, the cells were incubated at 37°C with 0.1 µg/ml KaryoMAX® Colcemid^TM^ (Life Technologies) for one hour, trypsinized, added to 6 ml of hypotonic solution (0.075 M KCl with HEPES) and fixed with methanol-acetic acid solution (3:1; Merck). The slides were placed in a water bath at 60°C, and drops of the cell suspension were placed on slides. Chromosomal preparations were submitted to the G-banding method using trypsin and Giemsa-staining. This protocol was based on protocols by Moralli et al. (2011) with modifications and Borgonovo et al. (2014) with modifications.

### Analysis and interpretation

The metaphases were digitally captured with a Leica DM Microscope (DM2000). LUCIA-KARYO (Laboratory Universal Computer Image Analysis LIM - Laboratory Imaging s.r.o) was used for the analysis. Whenever possible, 20 metaphases were analyzed. To be considered a clone, the same structural aberration or gain of the same chromosome had to be present in at least two metaphase cells, and the loss of a a chromosome must have been detected in at least three cells (McGowan-Jordan et al., 2016).

The statistical analysis was performed with IBM SPSS Statistics v.20.0 software (Armonk, NY; IBM Corp). The results were evaluated using the median, minimum and maximum values. Frequencies and percentages were defined for categorical variables. Fisher’s exact test was used to analyze the association between two categorical variables. Values of p<0.05 indicated statistical significance.

## Results

Ninety-seven samples from 38 iPSC lines between the 3rd and 34th passages were evaluated. A total of 632 metaphases were analyzed. Samples without metaphase accounted for 10.8% of all analyzed cases. The majority (71%) of samples showed a normal karyotype (excluding samples with less than 20 normal metaphases or those without metaphases).

The samples were separated into two groups. The first group was the disease group including cells from patients diagnosed with LQTS type 1 (n=6), LQTS type 2 (n=8), JLNS (n=2), ALS (n=3) and undetermined diagnoses of LQTS (n=2), totaling 21 samples. The second group was the nondisease group with 17 samples.

**Figure 1 -:**
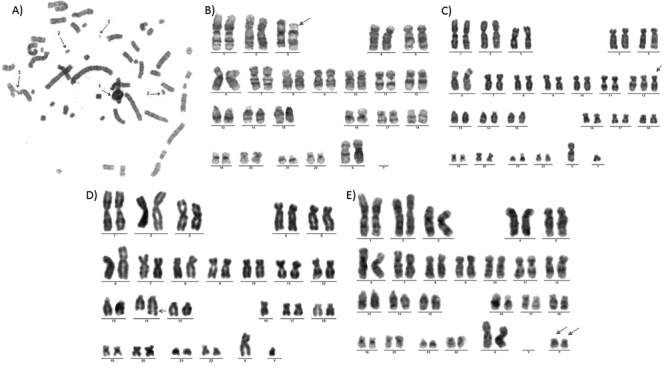
Cytogenetic analysis. A) Metaphase with ring chromosome (1), double minutes (2) and figure (3) (Sample IP028). B) Karyogram showing chromosome 3 inversion (Sample IP037). C) Karyogram showing chromosome 12 trisomy (Sample IP044). D) Karyogram showing additional material on the long arm of chromosome 14 (Sample IP056). E) Karyogram showing two marker chromosomes (Sample IP039).

Cytogenetic analysis demonstrated the presence of the following structural chromosomal alterations: acentric fragments, chromosomal fusions, premature centromere divisions, double minutes (dmin), radial figures, ring chromosomes, inversions, additional material and marker chromosomes, and the following numerical chromosomal alterations: polyploidies and trisomies (Figure 1). In the disease group, structural alterations were only observed in LQTS type 2 samples, and in the nondisease group, the samples derived from Wharton’s jelly presented both numerical and structural alterations, while those derived from blood presented only structural alterations.

### Nondisease and disease groups

Both the disease and nondisease groups presented alterations, with no significant difference between them (p=0.139, Fisher’s exact test).

The following percentages of altered samples were observed for each disease in the disease group: LQTS (n=14, frequency 21.4%), JLNS (n=2, frequency 0%) and ALS (n=3, frequency 0%); all of the cells were derived from peripheral blood.

Among the total samples from each group, 15.8% were altered in the disease group and 40% were altered in the nondisease group. Considering only the samples derived from peripheral blood in the nondisease group, 16.7% (n=6) were altered, while 62.5% (n=8) of samples obtained from Wharton’s jelly were altered.

Karyotype analysis in the different groups is shown in Table 1.

**Table 1 -:** Karyotype analysis in the different groups.

Patient number	Passage	Disease	Source	Karyotype
IP020	3	LQTS type 1	Peripheral blood	Without metaphases
IP023	4	LQTS type 1	Peripheral blood	46,XX[20]
IP031	3	LQTS type 1	Peripheral blood	46,XX[20]
IP033	5	LQTS type 1	Peripheral blood	46,XX[20]
IP036	3	LQTS type 1	Peripheral blood	46,XX[20]
IP050	6	LQTS type 1	Peripheral blood	46,XX[20]
IP016	11	LQTS type 2	Peripheral blood	Without metaphases
IP017	11	LQTS type 2	Peripheral blood	46,XY[3]
IP025	16	LQTS type 2	Peripheral blood	46,XX[20]
IP028	15	LQTS type 2	Peripheral blood	36~45,XY,~6dmin,+r1,+r2[6]/46,XY[14]
IP037	6	LQTS type 2	Peripheral blood	46,XX,inv(3)(p24q2?9)[6]/46,XX[14]
IP039	28	LQTS type 2	Peripheral blood	37~45,XX[9]/42~48,XX,+mar1,+mar2[11]
IP040	6	LQTS type 2	Peripheral blood	46,XY[20]
IP051	7	LQTS type 2	Peripheral blood	46,XX[21]
IP026	5	LQTS undetermined	Peripheral blood	46,XY[20]
IP019	3	LQTS undetermined	Peripheral blood	46,XY[2]
IP018	3	JLNS	Peripheral blood	46,XY[2]
IP030	4	JLNS	Peripheral blood	46,XY[30]
IP042	16	ALS	Peripheral blood - erythroblasts	46,XY[20]
IP052	12	ALS	Peripheral blood - erythroblasts	46,XY[20]
IP053	28	ALS	Peripheral blood - erythroblasts	46,XY[20]
IP021	3	No	Peripheral blood	Without metaphases
IP029	5	No	Peripheral blood	46,XX[20]
IP035	5	No	Peripheral blood	46,XX[30]
IP038	25	No	Peripheral blood	46,XY,inv(9)(p12q13)[20]
IP048	5	No	Peripheral blood	46,XY[20]
IP054	14	No	Peripheral blood - erythroblasts	46,XX[23]
IP055	20	No	Peripheral blood - erythroblasts	46,XY[20]
IP046	21	No	Peripheral blood - erythroblasts	46,XY[20]
IP041	12	No	Wharton’s jelly	46,XY[20]
IP043		No	Wharton’s jelly	Without metaphases
IP044	24	No	Wharton’s jelly	47,XY,+12[13]
IP045	13	No	Wharton’s jelly	92,XXXX[20]
IP047	14	No	Wharton’s jelly	46,XY[20]
IP056	23	No	Wharton’s jelly	46,XY,add(14)(q32)[13]
IP057	17	No	Wharton’s jelly	48~92,XXXX[14]
IP058	34	No	Wharton’s jelly	34~47,XY,+12[20]
IP059	29	No	Wharton’s jelly	46,XY[20]

### Differences based on iPSC sources

**Figure 2 -:**
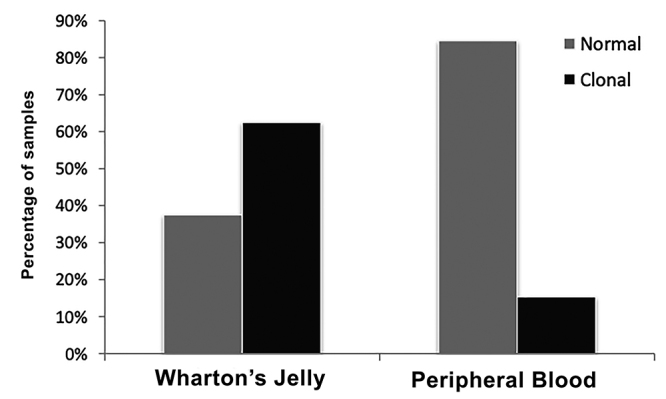
Percentage of cases in the nondisease group with normal results x clonal alteration in different sources.

The difference between the samples that presented clonal alterations and those that presented normal cytogenetics analyses in relation to the source are showed at Figure 2. This evaluation was performed only in the nondisease group, since among the individuals with diseases, there was no difference in the source (in all these cases, the source was peripheral blood). Samples without metaphases and less than 20 normal metaphases were excluded from this count.

### Influence of passage numbers

The mean number of passages of the samples that presented clonal alterations was different from that of samples that presented with normal karyotypes.

In samples derived from peripheral blood, the mean number of passages of cells with a normal karyotype was 9.18 (standard deviation=6.93), while that of abnormal cells was 18.5 (standard deviation=8.67). The medians were also different: 6 for normal cells and 20 for altered cells.

Samples derived from Wharton’s jelly also showed differences in the mean passage number. The passage number of samples with normal cells was 18.3 (standard deviation=7.58), and the passage number of samples with altered cells was 22.2 (standard deviation=7.13). The medians were also different: 14 for normal cells and 23 for altered cells.

### Karyotype before and after reprogramming

It was possible to perform karyotype analysis before and after reprogramming in two samples of mesenchymal stem cells derived from Wharton’s jelly. Each mesenchymal cell sample generated two iPSC samples (Table 2). A difference was observed between the results found before and after reprogramming in the two samples.

**Table 2 -:** Cytogenetic result before (mesenchymal) and after (iPSCs) reprogramming.

Mesenchymal sample	Mesenchymal result	iPSC samples	iPSC results
ME310	92,XXXX[4]/46,XX[29]	IP045	92,XXXX[20]
		IP057	48~92,XXXX[14]
ME311	46,XY[18]	IP044	47,XY,+12[13]
		IP058	34~47,XY+12[20]

## Discussion

The following chromosomal alterations were observed in this study: acentric fragments, chromosomal fusions, premature centromere divisions, double minutes, radial figures, ring chromosomes, polyploidies, inversions and trisomies. Both the disease and nondisease groups presented alterations. In the disease group, alterations were only observed in LQTS type 2 samples. In the nondisease group, the samples derived from Wharton’s jelly presented both numerical and structural alterations, while the samples derived from blood presented only structural alterations.

Trisomy 12 was observed in two samples, which is concerning because according to Ben-David et al. (2014), in cell culture, this alteration may result in an increased cell proliferation rate and, consequently, increased *in vivo* tumorigenicity, inducing teratomas. In addition, lineages with this alteration behave differently from those with a normal karyotype, showing a different sensitivity in drug tests (Ben-David *et al*., 2014). Therefore, the presence of trisomy 12 could be cause for exclusion of such samples for use in both transplants and disease models.

Other changes observed may be markers of oncogenesis, such as a tetraploid karyotype, which has been described in the transition from premalignant to malignant disease, suggesting that duplication of the genome may be a driver of tumorigenesis (Dewhurst et al., 2014). Furthermore, the presence of additional material on chromosome 14 was observed in one of our samples. Changes in 14q32, such as translocations and additional material, are observed in patients with lymphomas and lymphocytic leukemias (Chen et al., 2016; Veloza et al., 2019).

*Double minutes* are small, acentric and paired structures and were detected in a sample from a patient with LQTS type 2. The presence of dmins has been observed in patients with myelodysplastic syndrome, acute myelogenous leukemia or chronic myelomonocytic leukemia, and amplification of the *MYC* and *MLL* genes is observed in almost all cases (Huh et al., 2016). Future research with complementary molecular techniques could clarify whether the dmins observed after iPSC reprogramming could be copies of some of the genes used for reprogramming. The same sample also exhibited ring chromosomes in 6.7% of cells. Ring chromosomes arise after deletion or shortening of telomeric regions. Interestingly, shortening of telomeres associated with oxidative stress may cause genetic imbalances associated with ion channel defects in patients with LQTS (Yeh and Wang, 2016). Therefore, the presence of ring chromosomes may be a marker of LQTS, which is characterized by changes in sodium (Na^+^) and potassium (K^+^) ion channels (Nogueira et al., 2011). Nikitina et al. (2018) also reported the presence of ring chromosomes in iPSCs derived from skin fibroblasts; however, in this case, this chromosomal alteration was present before reprogramming because it was constitutional.

Chromosome 3 inversion, observed in a patient with LQTS, has previously been observed in patients with myelodysplastic syndrome and is often accompanied by alterations in other chromosomes. This inversion may be an indicator of evolution to acute myeloid leukemia and is therefore considered a chromosomal alteration associated with poor prognosis (Cui et al., 2011; Sun et al., 2011).

Chromosome 9 inversion, observed in one of our samples, is an inherited variant in 0.8 to 2% of the world population (Lee et al., 2010) and is not necessarily associated with neoplastic diseases.

Regarding the when alterations appear during cultivation, our results show that clonal alterations can be present in several passages because they were detected from P6 to P34. However, in samples analyzed from different donors, we observed that changes were more frequent in the late passages, as demonstrated by the Mann-Whitney nonparametric statistical test (p=0.003). The median number of passages of samples with alterations was 23, and the median number of passages of samples with normal results was six. Our results, together with data from the literature (Mayshar et al., 2010), show that aberrations can emerge in the initial passages with low frequency and can be detected only in the late passages. These data confirm the importance of cytogenetic tests during the cultivation of iPSCs, even if the cells have been cultivated for only a few passages.

Five of nine samples generated from Wharton’s jelly showed chromosomal aberrations. The only aberration found in the samples generated from peripheral blood was chromosome 9 inversion, which, as previously mentioned, is a variant commonly found in the population. Even when considering that chromosome 9 inversion may have developed during culture of iPSCs obtained from peripheral blood, we observed a significant difference between cells obtained from different sources (Fischer’s exact test, p=0.017), demonstrating an association between the source and the frequency of alterations. In this study, a greater frequency of chromosomal aberrations was observed in cells derived from Wharton’s jelly than in cells derived from peripheral blood.

## Conclusion

This study showed that, in iPSCs, there is a possibility that chromosomal aberrations, both clonal (26.4% of our samples) and nonclonal (20.5% of our samples), will emerge in several passages; these aberrations were detected from P6 to P34 and were more common in later passages. 

After reprogramming and cultivation, some abnormal clones can emerge, as in the ME311 case, or be selected and generate a completely altered lineage, as in the ME310 case. 

The following clonal chromosomal aberrations were detected in this study: chromosome 12 trisomy, which was previously reported in the literature; chromosome 3 inversion; chromosome 9 inversion; additional material on 14q; ring chromosomes; marker chromosomes; and dmins, which are being described now.

Many important chromosomal aberrations were detected, which demonstrated that G-banding is very useful for evaluation of genetic instability and has important repercussions for the application of iPSC lines.
